# Comprehensive analysis of the relationship between ubiquitin-specific protease 21 (USP21) and prognosis, tumor microenvironment infiltration, and therapy response in colorectal cancer

**DOI:** 10.1007/s00262-024-03731-4

**Published:** 2024-06-04

**Authors:** Haihang Nie, Yali Yu, Fan Wang, Xing Huang, Haizhou Wang, Jing Wang, Mi Tao, Yumei Ning, JingKai Zhou, Qiu Zhao, Fei Xu, Jun Fang

**Affiliations:** 1 Department of Gastroenterology, Zhongnan Hospital of Wuhan University, Wuhan, 430071 China; 2 Hubei Provincial Clinical Research Center for Intestinal and Colorectal Diseases, Wuhan, 430071 China; 3 Department of General Medical, Zhongnan Hospital of Wuhan University, Wuhan, 430071 China; 4 Department of Nephrology, Zhongnan Hospital, Wuhan University, Wuhan, 430071 China; 5 Hubei Key Laboratory of Intestinal and Colorectal Diseases, Zhongnan Hospital of Wuhan University, Wuhan, China

**Keywords:** Ubiquitin, USP21, Colorectal cancer, Tumor microenvironment, Immunotherapy, Prognosis

## Abstract

**Background:**

Ubiquitin-specific proteases family is crucial to host immunity against pathogens. However, the correlations between USP21 and immunosurveillance and immunotherapy for colorectal cancer (CRC) have not been reported.

**Methods:**

The differential expression of USP21 between CRC tissues and normal tissues was analyzed using multiple public databases. Validation was carried out in clinical samples through qRT-PCR and IHC. The correlation between USP21 and the prognosis, as well as clinical pathological characteristics of CRC patients, was investigated. Moreover, cell models were established to assess the influence of USP21 on CRC growth and progression, employing CCK-8 assays, colony formation assays, and wound-healing assays. Subsequently, gene set variation analysis (GSVA) was used to explore the potential biological functions of USP21 in CRC. The study also examined the impact of USP21 on cytokine levels and immune cell infiltration in the tumor microenvironment (TME). Finally, the effect of USP21 on the response to immunotherapy and chemotherapy in CRC was analyzed.

**Results:**

The expression of USP21 was significantly upregulated in CRC. High USP21 is correlated with poor prognosis in CRC patients and facilitates the proliferation and migration capacities of CRC cells. GSVA indicated an association between low USP21 and immune activation. Moreover, low USP21 was linked to an immune-activated TME, characterized by high immune cell infiltration. Importantly, CRC with low USP21 exhibited higher tumor mutational burden, high PD-L1 expression, and better responsiveness to immunotherapy and chemotherapeutic drugs.

**Conclusion:**

This study revealed the role of USP21 in TME, response to therapy, and clinical prognosis in CRC, which provided novel insights for the therapeutic application in CRC.

**Supplementary Information:**

The online version contains supplementary material available at 10.1007/s00262-024-03731-4.

## Introduction

Colorectal cancer (CRC) is the third most common cause of cancer mortality worldwide with more than 1.85 million cases and 850 000 deaths annually [1]. While there exists a plethora of treatments for colorectal cancer, including surgery, radiotherapy, chemotherapy, and immunotherapy, their efficacy has significantly improved over time. Nonetheless, long-term benefits for patients remain largely unchanged [2–4]. Immunotherapy has garnered substantial interest in cancer treatment research due to its noted advantages, such as high efficacy and minimal side effects. However, a considerable number of patients still exhibit resistance to immunotherapy, a phenomenon with multifaceted underlying reasons [4]. Consequently, the optimization of immunotherapeutic responsiveness stands out as an urgent and pivotal challenge to address.

Ubiquitin-specific proteases (USPs) family represents the most abundant category of deubiquitinating enzymes (DUBs) subfamilies [5]. An accumulating body of research suggests that USPs play a crucial role in modulating the efficacy of immunotherapy by regulating immune cell functions and responses within the tumor microenvironment (TME) [6–8]. The expression of certain USPs in tumor cells has been linked to tumor immune evasion and the mediation of resistance [8, 9]. USP21 is a member of the USP family, characterized by its C-terminal catalytic DUB domain. It serves as a nucleocytoplasmic shuttle protein, mediating the deubiquitination of RIPK1, FOXM1, STING, and histone H2A [10–14]. Located on chromosome 1q21, USP21 resides in a region housing various oncogenes such as MDM2 and creb314. The amplification of these genes is associated with poorer survival rates in several human cancers [15–18]. Moreover, USP21 has been implicated in the activation of many biological signaling pathways. It facilitates the activation of tumor stem cells via the Wnt signaling pathway and augments tumor therapeutic resistance by promoting the Hippo/YAP signaling pathway [19, 20]. Nonetheless, there is currently a lack of research on the prognostic significance of USP21 in colorectal cancer and its regulatory mechanisms within the TME.

In this study, we first evaluated the prognostic value of USP21 and its correlation with clinical characteristics in CRC and explored the potential biological functions and signaling pathways of USP21 in CRC growth and development. Then, the influence of USP21 on TME infiltration was analyzed. Finally, the responsiveness of USP21 to both immunotherapy and chemotherapy was assessed. We suggest that this study lays the groundwork for considering USP21 as a promising target for immunotherapeutic interventions in CRC.

## Methods

### Collection and preprocessing of CRC dataset

The TIMER database (https:// cistr ome. shiny apps. io/timer/) was used to analyze USP21 expression levels in pan-cancer data. Two independent patient cohorts with CRC were collected: GSE39582 was downloaded from the Gene-Expression Omnibus (GEO; https://www.ncbi.nlm.nih.gov/geo/) and TCGA-COAD (The Cancer Genome Atlas-Colon Adenocarcinoma) was downloaded from the UCSC Xena browser (https://xenabrowser.net/datapages/). In addition, the RNA-sequencing profile results of normal colon cohort of 307 samples were downloaded from Genotype-Tissue Expression Project (GTEx) (https://www.gtexportal.org/). The detailed information for all cohorts can be found in Table S1.

### Specimen collection

Fourteen CRC tissues and paired normal tissues were obtained from the Zhongnan Hospital of Wuhan University between January 2022 and September 2023 following patient informed consent. All patients were diagnosed by original histopathological detection and none of them received preoperative adjuvant chemotherapy or radiotherapy. Samples of the collected tissues were preserved in liquid nitrogen. The study protocol was approved by the Medical Ethics Committee of the Zhongnan Hospital of Wuhan University (grant no. 2024049).

### Cell culture and transfection

Two human CRC cell lines, SW480 and HCT116, were procured from the China Center for Type Culture Collection (Wuhan, China). The cells were cultured in Dulbecco’s modified Eagle’s medium (DMEM) (GIBCO, USA) containing 10% fetal bovine serum (FBS) (HyClone, USA), 100 U/ml penicillin, and 100 mg/ml streptomycin (Genom, China), at 37 °C with 5% CO_2_. siUSP21 and FAM-labeled siNC were obtained from Guangzhou RiboBio (Guangzhou, China) and transfected into the cancer cell lines using Lipofectamine 3000 (Invitrogen, USA). Transfection efficiency of siRNA with Lipofectamine 3000 was evaluated using a fluorescence microscope (Olympus U-RFL-T, Japan) 24 h post-transfection, and the knockdown efficiency of siUSP21 was further confirmed through quantitative real-time polymerase chain reaction (qRT-PCR) and Western blot experiments.

### RNA extraction and qRT-PCR

The total RNA in tissues and cells was extracted using Trizol reagent (Invitrogen, USA), followed by reverse transcription with the TOYOBO ReverTra Ace kit (TOYOBO, Japan). qRT-PCR was conducted utilizing UltraSYBR mixture (Cwbio, China) on a Roche LightCycler 96 PCR system. mRNA expression levels were quantified via qRT-PCR using the Biorad CFX system (Biorad, USA), with GAPDH serving as the internal control gene. Primers were synthesized by TSINGKE Biological Technology (Wuhan, China), and their sequences were as follows: GAPDH Forward: 5′-GGAGCGAGATCCCTCCAAAAT-3′and Reverse: 5′-GGCTGTTGTCATACTTCTCATGG-3′ and USP21 Forward: 5′-TGCTCCCCACTTTCGTTGAT-3′ and Reverse: 5′-CTGGGGGCAAATGGTAGCTT-3′. The mRNA expression levels were determined using the comparative CT method (2-ΔΔCT), and all experiments were performed with three independent biological replicates.

### Protein extraction and western blotting

Total protein in CRC lines was extracted using RIPA lysis buffer (Boyotime, China) according to the reagent instructions. Western blotting was performed with the specific antibody, USP21 (1:1000, Proteintech, China, 17,856-1-AP) and GAPDH (1:1000, Proteintech, China, 60,004-1-Ig).

### Immunohistochemistry (IHC)

CRC and normal tissues were fixed in 10% neutral-buffered formalin (Sigma-Aldrich, USA) and embedded in paraffin. Tissue sections were sliced from paraffin blocks into 3-μm-thick slices. Heat-mediated antigen retrieval was performed using EDTA buffer pH 9.0. Primary antibodies targeting USP21 (1:200, Proteintech, China, 17,856-1-AP), PD-L1 (1:200, Proteintech, China, 28,076-1-AP), CXCL11 (1:200, Proteintech, China, 10,707-1-AP), and CXCL10 (1:200, Proteintech, China, 10,937-1-AP) were employed for immunostaining. Image analysis was conducted using Image J, and relative expression was determined accordingly. Finally, we compared the expression levels of CXCL10, CXCL11, and PD-L1 in two cancer tissues with the most significant differences in USP21 expression.

### CCK-8 assay

Cell viability was detected using CCK-8 kit (Dojindo Molecular Technologies, Japan). 1000 cells transfected with siUSP21 or siNC were seeded per well in 96-well plates, with each condition having five replicate wells. After incubation for 2 h, CCK-8 reagent was added to the wells along with the cell culture medium. Absorbance at 450 nm was measured using a microplate reader (ELX-800; BioTek, USA), and the growth curve was constructed based on the optical density (OD) values.

### Colony formation assay

Following siRNA transfection for 24 h, 1000 cells were seeded into a six-well culture plate and incubated at 37 °C with 5% CO_2_. Four days later, a subsequent transfection was conducted on these cells, and visible colonies were observed after 10 days. The colonies were fixed with 4% paraformaldehyde and stained with crystal violet. The number of colonies consisting of at least 50 cells was quantified.

### Wound-healing assay

HCT116 or SW480 cells were seeded, and scratch wounds were made when cell confluence reached approximately 80% at approximately 48 h post-transfection. Subsequently, the cultures were incubated in serum-free medium for 24 h. Following this, three random visual fields were selected from each scratch wound and observed under a microscope. All experiments were conducted in triplicate.

### Evaluate the prognostic and clinicopathological value of USP21

Univariate Cox (uni-Cox) and multivariate Cox (multi-Cox) regression analyses were used to identify independent risk factors linked with USP21, encompassing USP21 and diverse clinicopathological characteristics, such as age, gender, TCGA type, stage, grade, and TNM stage. The MEXPRESS database (https://mexpress.be/) was used to investigate the correlation between USP21 and DNA methylation. Kaplan–Meier (KM) survival analysis was conducted on the overall survival (OS), progression-free interval (PFI), and disease-specific survival (DSS) using the “survival” and “survminer” R packages.

### Differentially expressed genes (DEGs) analysis

The TCGA-COAD patients were divided into high and low USP21 expression groups using the median USP21 as a cutoff. The empirical Bayesian approach of the “limma” R package was employed to determine DEGs [21]. The significance criteria for identifying DEGs were set as adjusted *p* value < 0.05 and |log2(fold change)|> 0.65.

### Biological function, pathway annotation and gene set variation analysis (GSVA)

The "GSVA" R package was used for GSVA analysis to explore the differences in biological processes between the high and low USP21 groups. The gene set of “c2.cp.kegg medicus.v2023.2.Hs.symbols” was downloaded from MSigDB. A fold change of |log2(fold change)|> 0.25 and an adjusted *p* value of < 0.05 were considered statistically significant.

### Correlation between USP21 and TME

Analysis of correlation between USP21 and TME be referred to our previous study [22, 23].

### Response of immunotherapy and drug susceptibility analysis

Six immunotherapy cohorts were downloaded from the GEO database. In addition, an immunotherapy cohort of melanoma was collected from the supplementary files of Nathanson’s study [24]. The detailed information can be found in Table S1. Subsequently, stratified analysis was conducted on the immune therapy response. The "pRRophetic" R package was used to calculate the semi-inhibitory concentration (IC50) values of commonly used chemotherapeutic drugs.

### Statistical analysis

All analyses were performed using R software version 4.1.3 and GraphPad Prism 8.0 (GraphPad, San Diego, CA, USA). The Wilcoxon or Kruskal–Wallis test was used for comparing two or multiple groups, respectively. If the data were non-normally distributed, the Mann–Whitney or Dunn's test was employed. All statistical significance was defined as *p* < 0.05.

## Results

### Higher USP21 inferred a worse prognosis for CRC

**Fig. 1:**
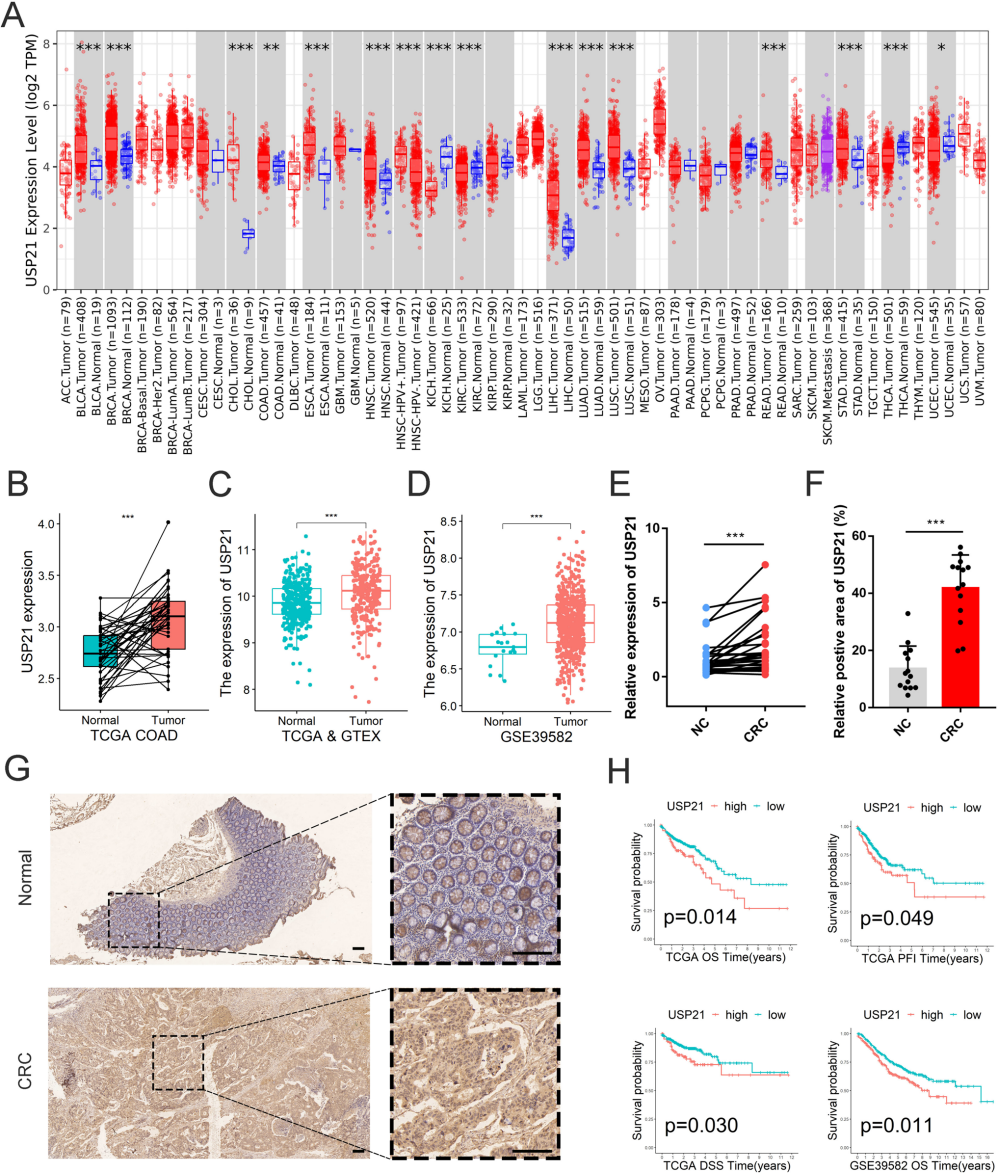
USP21 expression analysis in CRC. **A** USP21 expression in normal colon and CRC tissues in pan-cancer data from the TIMER website. **B** Comparison of USP21 expression between adjacent non-cancerous and cancerous tissues in CRC patients from the TCGA database. **C** Differential expression of USP21 between normal colon and CRC samples from TCGA and GTEx.** D** Differential expression of USP21 between normal colon and CRC samples from GSE39582.** E** USP21 expression in 14 pairs of CRC tissues and normal tissues detected by qRT-PCR**. F** Quantification of IHC positive areas of USP21 in normal and CRC tissues. **G** Representative cases of USP21 expression normal colon and CRC tissues. Scale bar, 50 mm. **H** Kaplan–Meier analysis of USP21 expression based on OS, DFI, and DSS in the TCGA-COAD and GSE39582. *p < 0.05, **p ≤ 0.01, and ***p ≤ 0.001; CRC, colorectal cancer

In a comprehensive pan-cancer analysis, USP21 emerged as significantly upregulated across various gastrointestinal malignancies, including colon, esophageal, liver, and stomach cancers (Fig. 1A). Validation in 41 pairs of colon cancer samples from the TCGA-COAD cohort confirmed a pronounced elevation of USP21 expression specifically in CRC (Fig. 1B). This finding was further substantiated by analyses of normal samples from the GTEx cohort and external validation from the GSE39582 cohort (Fig. 1C-D). Subsequent mRNA and immunohistochemical analyses conducted in 14 collected CRC patient tissues consistently demonstrated a marked upregulation of USP21 at both the mRNA and protein levels within CRC (Fig. 1E-G; Fig. S1). The detailed pathological information can be found in Table S2. To elucidate the prognostic significance of USP21 expression levels in CRC patients, Kaplan–Meier survival analysis was performed, revealing inferior OS, PFI, and DSS in patients with high USP21 expression compared to those with low expression (Fig. 1H). Thus, our findings suggest that the upregulation of USP21 expression may serve as a potential prognostic marker in CRC patients, warranting further investigation.

### Association between clinicopathological characteristics and USP21

**Fig. 2:**
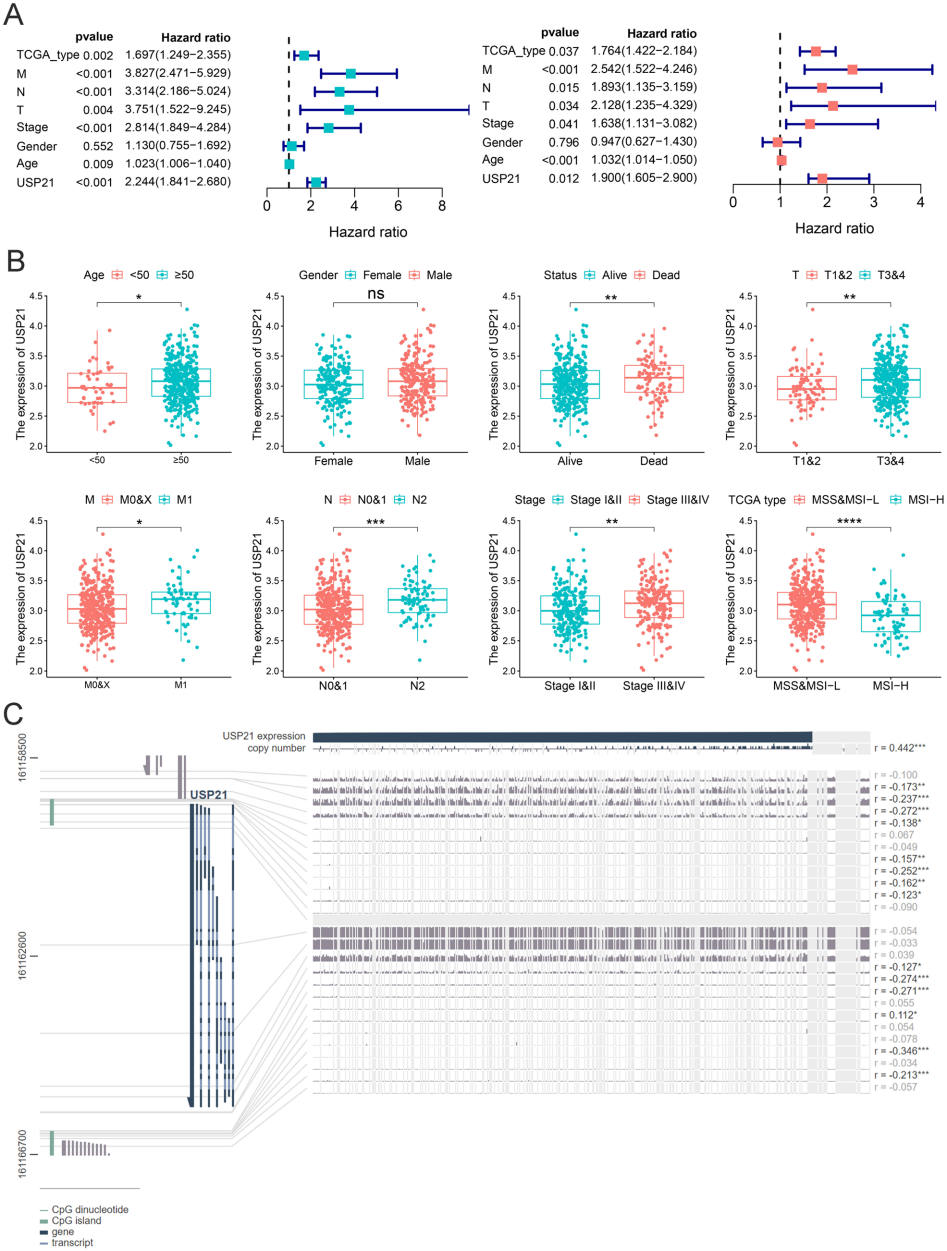
USP21 expression prognostic and clinicopathological value analysis. **A** Uni-Cox and multi-Cox regression analyses of clinical factors and USP21 expression with OS. Hazard ratio > 1 represented risk factors for survival and hazard ratio < 1 represented protective factors for survival.** B** Analysis of USP21 expression in different subtypes of CRC tissues from TCGA database. **C** The correlation of USP21 expression and DNA methylation level in MEXPRESS-COAD cohort. *p < 0.05, **p ≤ 0.01, and ***p ≤ 0.001; CRC, colorectal cancer; uni-Cox, univariate Cox; multi-Cox, multivariate Cox

We assessed the impact of high USP21 on the prognosis and clinical characteristics of CRC using the TCGA-COAD cohort. Initially, uni-Cox regression analysis revealed correlations between poor prognosis in CRC and factors such as TCGA subtype (HR = 1.697, 95%CI = 1.249–2.355, *p* = 0.002), TNM (T: HR = 3.751, 95%CI = 1.522–9.245, *p* = 0.004; N: HR = 3.314, 95%CI = 2.186–5.024, *p* < 0.001; M: HR = 3.827, 95%CI = 2.471–5.929, *p* < 0.001), pathological stage (HR = 2.814, 95%CI = 1.849–4.284, *p* < 0.001), age (HR = 1.023, 95%CI = 1.006–1.040, *p* = 0.009), and USP21 expression (HR = 2.244, 95%CI = 1.841–2.680, *p* < 0.001). Subsequent multi-Cox regression analysis confirmed that TCGA subtype (HR = 1.764, 95%CI = 1.422–2.184, *p* = 0.037), TNM (T: HR = 2.128, 95%CI = 1.235–4.329, *p* = 0.034; N: HR = 1.893, 95%CI = 1.135–3.159, *p* = 0.015; M: HR = 2.542, 95%CI = 1.522–4.246, *p* < 0.001), pathological stage (HR = 1.638, 95%CI = 1.131–3.082, *p* = 0.041), age (HR = 1.032, 95%CI = 1.014–1.050, *p* < 0.001), and USP21 expression (HR = 1.900, 95%CI = 1.605–2.900, *p* = 0.012) independently acted as risk factors for CRC (Fig. 2A). Further analysis unveiled high USP21 was associated with poorer clinical pathological staging, higher TNM staging, and MSS/MSI-L status. These findings suggest that high USP21 signifies increased tumor malignancy and poorer prognosis (Fig. 2B). Additionally, alterations in USP21 expression showed significant correlations with multiple methylation sites, implying the potential role of methylation in regulating USP21 expression in colorectal cancer and impacting tumor prognosis (Fig. 2C).

### USP21 promotes cell proliferation and migration of CRC cells

**Fig. 3:**
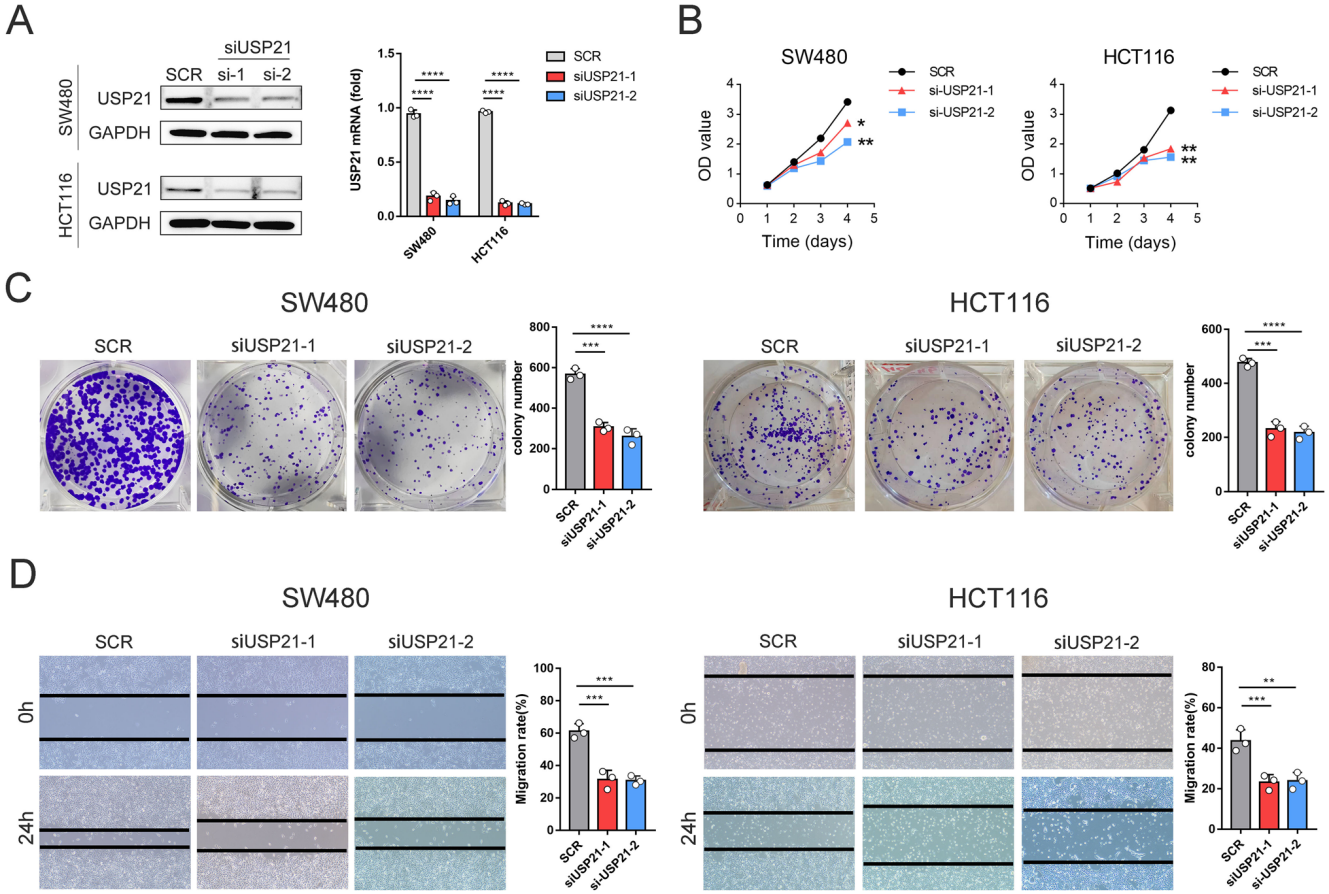
Effects of USP21 knockdown on CRC cell growth in vitro. **A** qRT-PCR and western blot analysis of siUSP21 efficiency in HCT116 and SW480 cell lines. **B** Cell proliferation after the knockdown of USP21 in HCT116 and SW480 cell lines was measured by the CCK-8 assay. **C** The colony formation assay was performed to assess the cell proliferation capability after the USP21 knockdown in HCT116 and SW480 cell lines. **D** Typical images of wound-healing assay of CRC cells modified by siUSP21 at 0, 24 h. *p < 0.05, **p ≤ 0.01, ***p ≤ 0.001, and ****p ≤ 0.0001; CRC, colorectal cancer

To further investigate the biological impact of USP21 in CRC, we employed siRNAs (siUSP21-1 and siUSP21-2) to silence USP21 expression in CRC cell lines. We assessed the transfection efficiency through both qRT-PCR and western blotting (Fig. 3A). Subsequently, we examined the effects of USP21 silencing in the proliferation and migration of CRC cells. Results from CCK-8 assays and colony formation assays revealed a significant inhibition of CRC cell proliferation following silence of USP21 (Fig. 3B-C). Wound-healing assays further demonstrated a notable suppression of CRC cell migration following silence of USP21 (Fig. 3D). Quantitative and statistical analyses of colony formation and wound-healing percentages, conducted over three experimental replicates, underscored significant differences between the siRNA control group and the siUSP21 group. Collectively, these findings underscore the role of USP21 in promoting CRC cell proliferation and migration.

### Identification of DEGs and GSVA in CRC

**Fig. 4:**
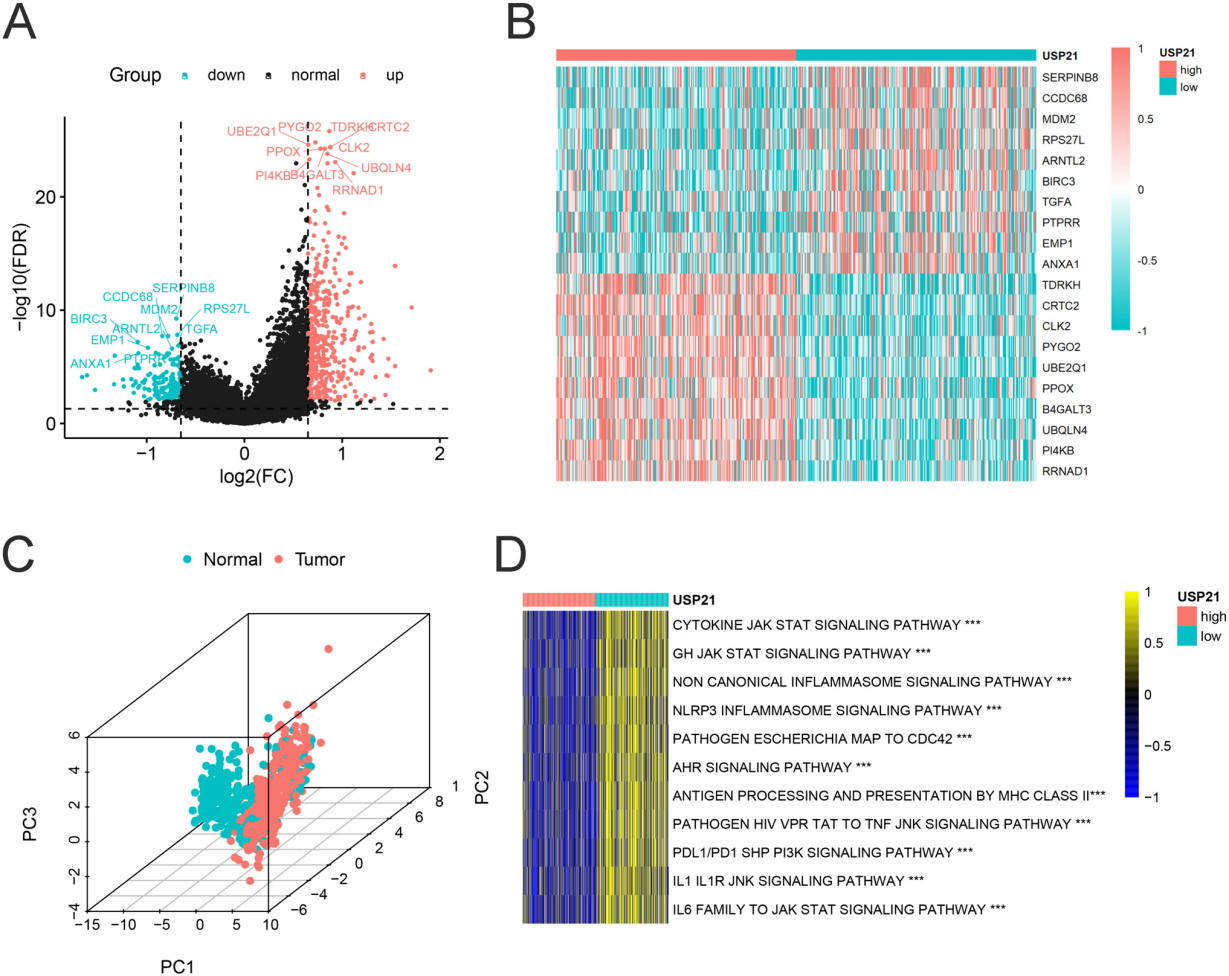
Enrichment analysis of USP21 biological function in the TCGA‑COAD cohort. **A** Volcano plot of DEGs between high and low USP21 group. **B** Heat map showed the top ten genes in both upregulated and downregulated DEGs. **C** The difference of expression levels of DEGs between normal colon and CRC. **D** GSVA showed the activation status of biological pathways in different USP21 expression groups using KEGG gene sets from MSigDB database. *p < 0.05, **p ≤ 0.01, and ***p ≤ 0.001; CRC, colorectal cancer; DEGs, differentially expressed genes

A total of 2221 DEGs were identified between high and low USP21 expression groups, comprising 530 downregulated genes and 1691 upregulated genes (Fig. 4A; Table S3). The top ten genes in both upregulated and downregulated DEGs demonstrated remarkable discriminatory capacity between high and low USP21 expression groups (Fig. 4B). Principal component analysis (PCA) further confirmed the effective discrimination of these DEGs between tumor and normal tissues (Fig. 4C). Notably, PYGO2, UBQLN4, and ANXA1 have been associated with responsiveness to tumor immune checkpoint blockade (ICB) therapy [25–27], while B4GAL and MDM2 are implicated in regulating CD8 + T cell function and tumor growth [28, 29]. Additionally, UBE2Q1, ARNTL2, CLK2, and EMP1 have been shown to participate in modulating the TME in various tumors [30–33]. Further exploration of potential biological pathways related to USP21 was conducted through GSVA using these DEGs. Enrichment analysis revealed several pathways associated with immune activation enriched in the low USP21 group, including the cytokine JAK-STAT signaling pathway, NLRP3 inflammasome signaling pathway, and non-canonical inflammasome signaling pathway. Moreover, the PD-L1/PD-1 SHP-PI3K signaling pathway was also enriched in the low USP21 group, suggesting a potential benefit of ICB therapy in tumors with low USP21 (Fig. 4D; Table S4). In summary, the differential expression of USP21 is intricately linked to the TME in CRC.

### Relationship of USP21 with immune system and TME

**Fig. 5:**
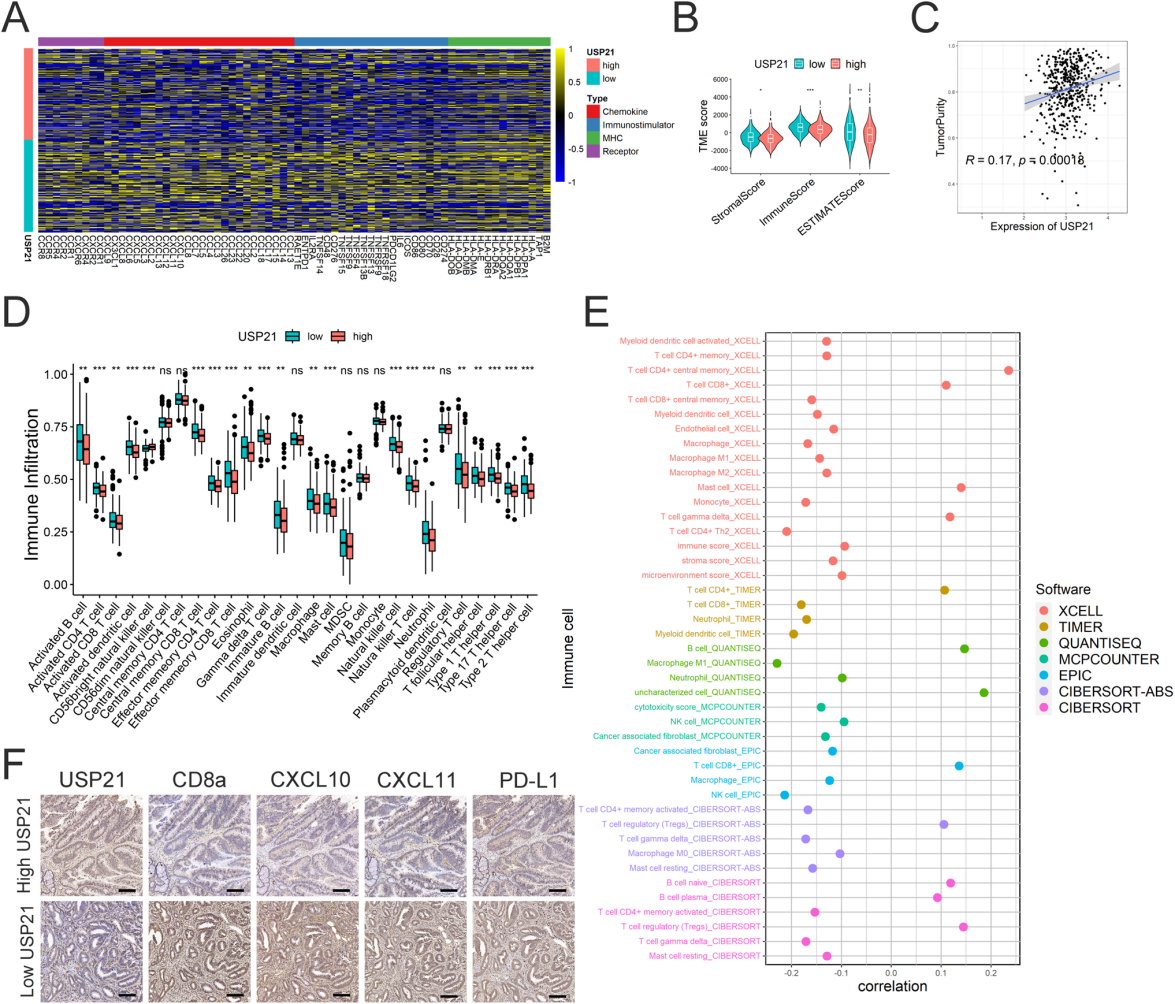
USP21 expression associated with the immune system and tumor microenvironment infiltration. **A** Expression levels of four immunomodulators (MHC, immunostimulators, chemokines and receptors) patients with CRC of high and low USP21 groups. **B** The comparison of stromal, immune, and ESTIMATE score between different USP21 expression groups. **C** Correlation between USP21 expression and tumor purity using the ESTIMATE algorithm. **D** Correlations between USP21 expression and 28 TIICs levels. **E** Correlation between USP21 and TIICs levels calculated using seven independent algorithms. **F** Representative photographs show the intratumoral expression of CD8a, CXCL10, CXCL11, and PD-L1 in samples with high USP21 and in samples with low USP21 in the same fields, on serial sections in CRC. Scale bar, 100 mm. *p < 0.05, **p < 0.01, ***p < 0.001, ns (*p* > 0.05); CRC, colorectal cancer; TIICs, tumor-infiltrating immune cells

**Fig. 6:**
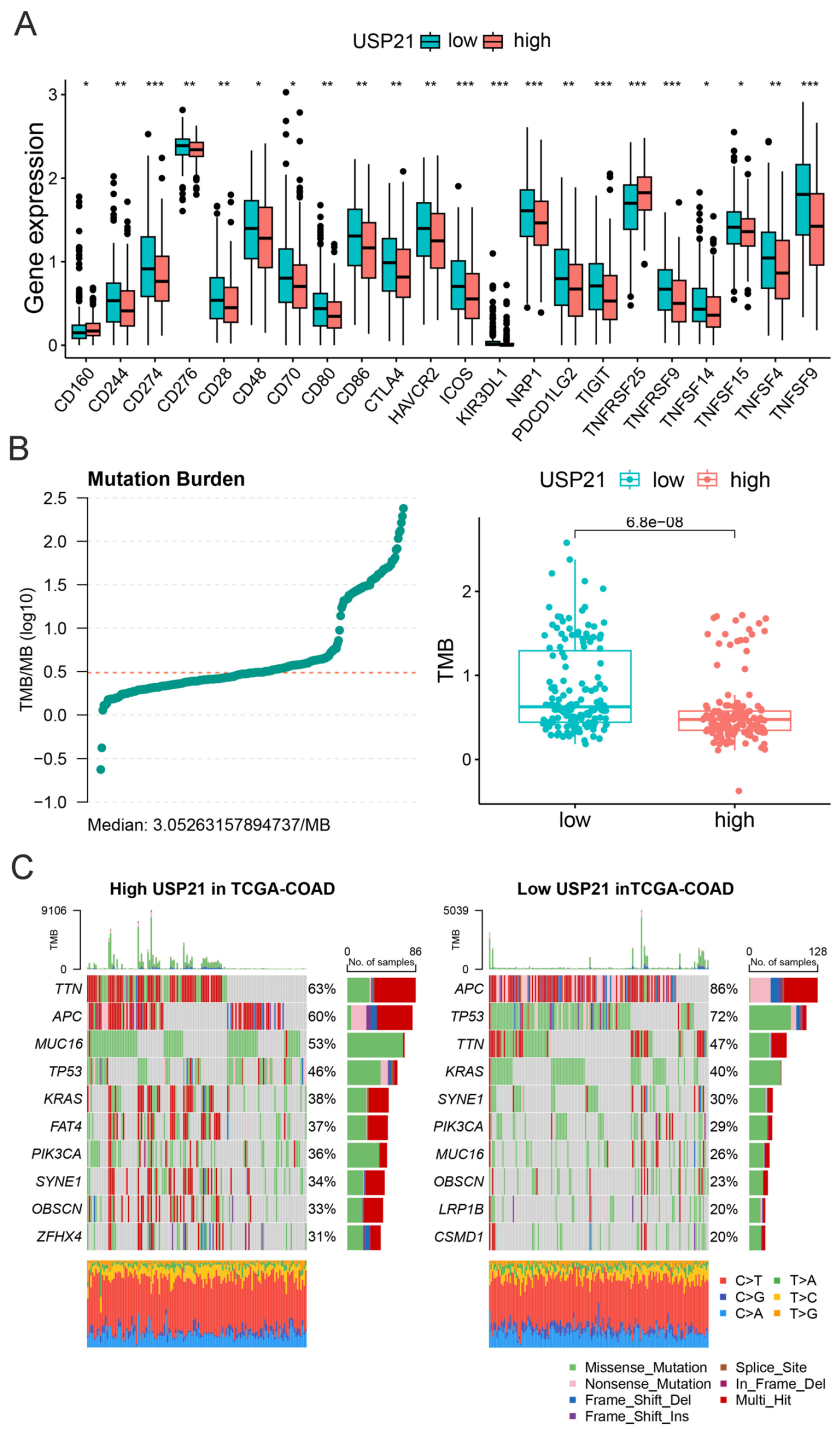
USP21 expression associated with immune checkpoints and genetic alterations in CRC. **A** Expression of immune checkpoints in high and low USP21 groups. **B** Analysis of correlation between USP21 expression with TMB level in the TCGA-COAD cohort. **C** Top 10 mutated genes were illustrated in CRC with high USP21and in CRC with low USP21. *p < 0.05, **p ≤ 0.01, and ***p ≤ 0.001

A comprehensive investigation was undertaken to evaluate the immunological role of USP21. Initially, it was observed that high USP21 resulted in a significant downregulation of immune regulators, including MHC molecules, immune stimulators, chemokines, and receptors, indicating a potential suppression of the tumor immune system (Fig. 5A). Moreover, it was noted that immune scores, stromal scores, and ESTIMATE scores were markedly reduced in the high USP21 group (Fig. 6B), with a positive correlation observed between tumor purity and USP21 (Fig. 6C). Analysis of the relationship between USP21 and TIICs revealed that nearly all immune cells were downregulated in the high USP21 group (Fig. 5D). Additionally, infiltration levels of TIICs were assessed using seven independent algorithms, yielding results consistent with our previous findings. Infiltration levels of CD8 + T cells, CD4 + T cells, neutrophils, and macrophages exhibited negative correlations with USP21 expression, while the microenvironment score calculated by the xCell algorithm showed a positive correlation with USP21 expression (Fig. 5E). Considering the crucial role of CD8 + T cells in anti-tumor immunity and their correlation with USP21 expression levels, immunohistochemical analysis was performed on collected colon cancer tissues, revealing a significant increase in CD8a (a characteristic marker of CD8 + T cells) in tissues with low USP21. Furthermore, CXCLs play pivotal roles in recruiting, migrating, and activating immune cells, especially CXCL10 and CXCL11 [34]. Consistently, in our tissue cohort, elevated expression of CD8a, CXCL10, and CXCL11 was observed in tissues with low USP21 expression (Fig. 5F). These findings collectively underscore the strong association between upregulated USP21 expression and TME suppression in CRC.

Furthermore, anti-PD-1/PD-L1 therapy has emerged as a prominent approach in cancer immunotherapy in recent years. Numerous studies have indicated that blocking the activation of the PD-1/PD-L1 pathway helps to reduce the immune evasion of tumor cells from CD8 + T cells. In our tissue cohort, we observed a negative correlation between USP21 and PD-L1 (Fig. 5F), suggesting the feasibility of anti-PD-1/PD-L1 therapy in combating CRC with low USP21.

### Low expression of USP21 is beneficial to immunotherapy for CRC

We sought to delve deeper into the potential factors influencing the efficacy of immunotherapy, focusing on immune checkpoints and genetic predisposition. Initially, we conducted a preliminary analysis of the relationship between USP21 and immune checkpoint expression, revealing a significant negative correlation with common immune checkpoints such as PD-L1, CTLA4, CD28, and TIGIT (Fig. 6A). Subsequently, we calculated the tumor mutational burden (TMB) scores in the TCGA-COAD cohort and observed a significantly higher TMB in low USP21 group compared to the high USP21 group (Fig. 6B). Waterfall plots exhibited a higher frequency of mutations in P53 and APC in low USP21 group (Fig. 6C). These findings collectively suggest that tumors with high USP21 may exhibit enhanced susceptibility to immunotherapy compared to those with low USP21.

### Immunotherapy and drug susceptibility analysis

**Fig. 7:**
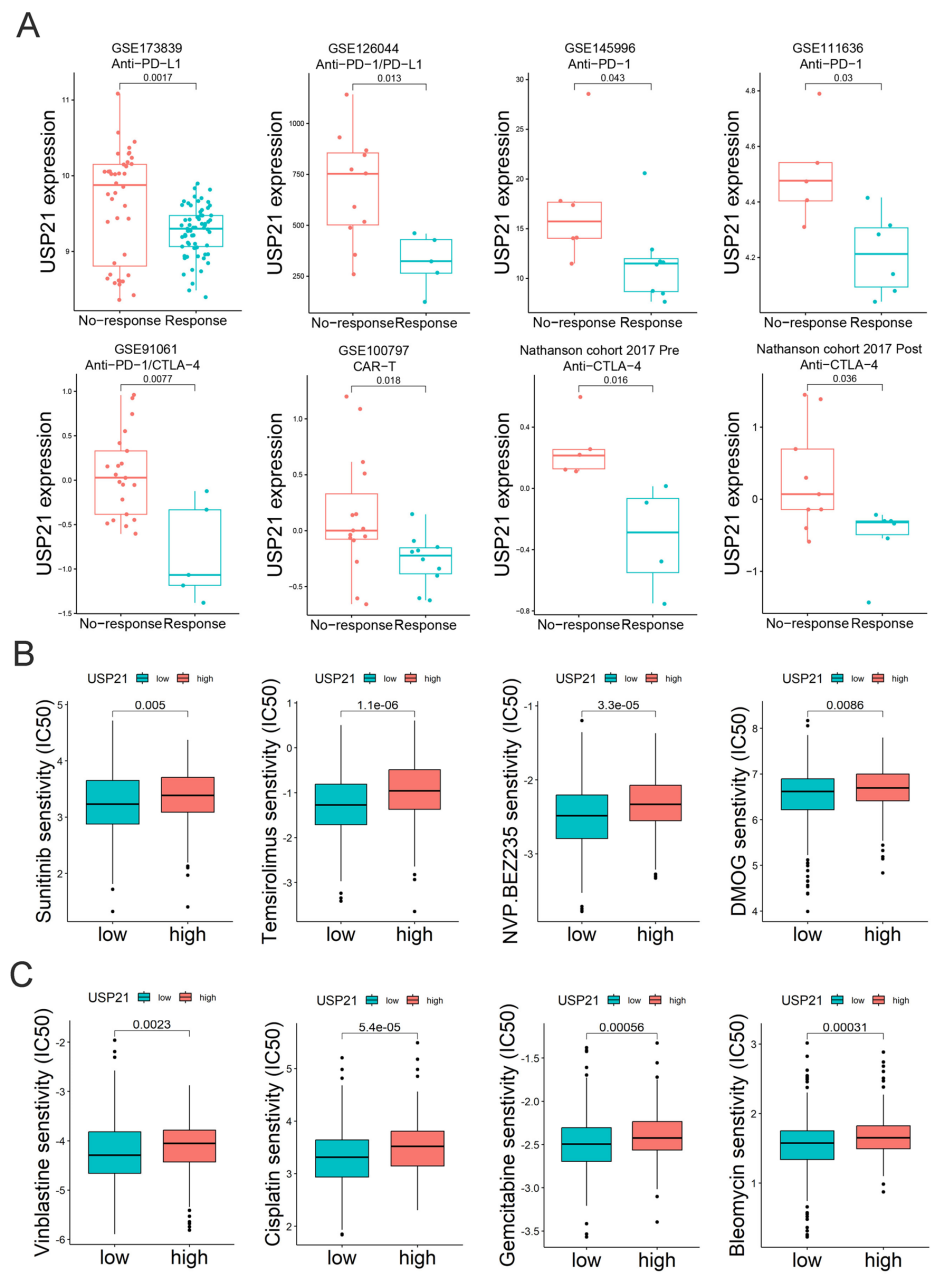
The comparison of response to immunotherapy and drug susceptibility between high and low USP21 groups. **A** Analysis of USP21 expression differences on responsiveness to different immunotherapies. **B** Influence of USP21 expression differences on targeted drug IC50. **C** Influence of USP21 expression differences on traditional chemotherapy IC50. *p < 0.05, **p ≤ 0.01, and ***p ≤ 0.001; IC50, half maximal inhibitory concentration

To ascertain the influence of USP21 in CRC on immunotherapy, we conducted validation within cohorts of tumor patients undergoing diverse treatment regimens. Results revealed that patients with low USP21 not only significantly benefited from anti-PD-L1/PD-1 therapy but also demonstrated notable treatment responsiveness to anti-CTLA4 and CAR-T (Fig. 7A). Additionally, we evaluated targeted therapy and conventional chemotherapy within the TCGA-COAD cohort. Compared with the high USP21 group, patients in the low USP21 group had lower IC50 to targeted drugs, such as sunitinib, temsirolimus, DMOG, and NVP.BEZ235, indicating that CRC with low USP21 was more sensitive to these drugs (Fig. 7B). Similarly, CRC with low USP21 displayed heightened sensitivity to traditional chemotherapy, including alkylating agents such as cisplatin, Vinblastine, antibiotics like bleomycin, and antimetabolites such as gemcitabine (Fig. 7C). In summary, this study suggests that USP21 has significant prognostic value for immunotherapy and chemotherapy.

## Discussion

DUBs play a pivotal role in altering protein status by removing ubiquitin molecules from substrates, thereby dynamically regulating protein quantity and activity within cells [35]. Studies have shown that USPs, a class of DUBs, represent the largest subfamily involved in regulating various tumor TME [6–8]. USP7 has been shown to foster tumor growth by modifying the immunosuppressive properties of Foxp3 + Treg cells [36]. USP14 inhibition can attenuate IDO1 protein levels, bolster CD8 + T cell infiltration, and reverse immune tolerance [37]. In this study, we uncovered for the first time that high USP21 promotes colorectal cancer (CRC) cell proliferation and progression, correlates with adverse patient prognosis, induces immunosuppression in TME, and confers resistance to immunotherapy. These findings underscore the potential of USP21, akin to other members of the USP family, in modulating the TME and position it as a promising target for immunotherapy.

Tumor molecular classification has emerged as a key focus in recent years. This approach not only distinguishes tumor subtypes but also offers insights into tumor progression and prognosis, facilitating personalized treatment strategies and predicting treatment response [38]. Proficient mismatch repair or microsatellite stability (pMMR/MSS) stands out as a robust predictor for immune checkpoint therapy in CRC. Our prior study has revealed that pMMR/MSS CRC displays immunogenic heterogeneity, indicating a tendency toward immunotherapy tolerance. Meanwhile, GBP2 has been identified as a prognostic marker for pMMR/MSS CRC patients, influencing both prognosis and immune response [34]. The BRAF V600E mutation, present in over 90% of BRAF mutations, affects approximately 10% of metastatic CRC cases, with an incidence ranging from 5 to 21%. In advanced CRC, the BRAF V600E mutation can be utilized for predicting OS in patients undergoing hepatic metastasectomy [39]. Similarly, our study underscores the utility of USP21 in CRC molecular classification. USP21 exhibits high expression in MSS/MSI-L subtype CRC and correlates with low differentiation and lymphatic metastasis. Furthermore, high USP21 corresponds to poorer prognosis and immune therapy resistance in CRC patients. Thus, delving deeper into the molecular and functional mechanisms of USP21 in CRC is paramount for elucidating its significance in molecular subtyping research.

In order to explore the underlying molecular mechanisms governing CRC progression regulated by USP21, we conducted a comprehensive analysis. Using mRNA sequencing data from public databases, we performed differential expression analysis, identifying molecules exhibiting pronounced expression variances as potential downstream targets of USP21. Notably, a majority of these molecules are implicated in regulating TME [25–33]. Functional enrichment analysis showed functional pathways associated with immune activation that correlate with low USP21. The findings collectively indicate that USP21 serves as a significant regulator of TME in CRC.

Our investigation into the interaction between USP21 and TME demonstrated the heightened malignancy and poorer prognostic outcomes observed in CRC with high USP21, from the perspective of tumor immunity. High USP21 in CRC engenders an immunosuppressive TME, thwarting the immune system's anti-tumor efficacy. Notably, CD8 + T cells represent crucial effectors against tumors [18, 33, 34]. Nevertheless, as of present, there remains a dearth of studies revealed the association between USP21 and CD8 + T cell in CRC. Our findings showed a marked reduction in CD8 + T cell infiltration in CRC with high USP21, accompanied by diminished expression of chemokines crucial for CD8 + T cell recruitment. Studies have revealed that USP21 can bind to phosphorylated STAT1, thereby upregulating FOXP3. This process enhances the immunosuppressive function of Treg cells within TME while diminishing the anti-tumor efficacy of CD8 + T cells [40, 41]. Therefore, we advocate for further exploration of the intricate mechanisms through which USP21 modulates the TME and CD8 + T cell dynamics via Treg cells in CRC, as a critical avenue for future research.

The effectiveness of tumor therapy is an inevitable topic. In recent years, immunotherapy has undoubtedly made great breakthroughs in tumor treatment. An essential molecular mechanism mediating immune evasion is the interaction between tumor cells expressing PD-L1 and PD-1 on the surface of CD8 + T cells [42]. In this study, immunohistochemical analysis revealed not only a diminished infiltration of CD8 + T cells but also reduced PD-L1 expression in CRC with high USP21. This suggests a potential reduced sensitivity of CRC with high USP21 to anti-PD-1/PD-L1 therapy. Meanwhile, CRC with low USP21 exhibited higher TMB, including TP53/KRAS mutations. Studies have shown that TP53/KRAS mutations can upregulate tumor PD-L1 expression, leading to more pronounced responses to PD-1/ PD-L1 inhibitors in patients [43]. Consistent with these findings, our analysis of the immunotherapy cohort confirmed that patients with low USP21 showed high responsiveness to anti-PD-1/PD-L1 therapy. Furthermore, patients with low USP21 also showed potential benefits from targeted therapy, conventional chemotherapy, and other immunotherapy such as anti-CTLA4 and CAR-T therapy. As previously mentioned, this correlation may be attributed to the immunosuppressive role of USP21 within TME. As reported, USP8 inhibition promotes K63-linked ubiquitination of PD-L1, decreasing its degradation while boosting immune response and MHC-I expression. Utilizing USP8 inhibitors can enhance anti-tumor efficacy of PD-1/PD-L1 blockade [8]. Thus, as part of USPs family, we suggest that USP21 holds promise as a potential immunotherapeutic target. Combining USP21 inhibition with immunotherapy, particularly ICB therapy, may enhance the anti-tumor effects.

Although the results of this study generally met expectations, there were still some limitations. TME analysis primarily relied on public databases. Due to the current scarcity of adequately high-quality fresh CRC samples, techniques such as flow cytometry and single-cell sequencing were not feasible. Additionally, the establishment of cellular or animal models to validate the impact of USP21 on TME remains pending. Furthermore, the creation of an immunotherapy cohort comprising CRC patients is imperative to further explore the influence of USP21 on immunotherapy efficacy and its underlying biological regulatory mechanisms.

In conclusion, this study reveals the association between upregulated USP21 expression and poor prognosis and clinical characteristics of CRC. Moreover, high USP21 expression to induce immune suppression potentially underpins the diminished responsiveness of CRC to immunotherapy. These findings highlight the significant clinical relevance of USP21 and offer novel insights into its role in CRC immunotherapy.

### Supplementary Information

Below is the link to the electronic supplementary material.

Supplementary file1 (DOCX 6376 KB)

Supplementary file2 (XLSX 738 KB)

## Data Availability

All public datasets enrolled in this study could download from GEO database (https://www.ncbi.nlm.nih.gov/geo/) and the UCSC Xena browser (https://xenabrowser.net/datapages/). All data generated or analyzed during this study are included in the supplementary information files of this article.
