# Supplementary material for: Comprehensive analysis of the relationship between ubiquitin-specific protease 21 (USP21) and prognosis, tumor microenvironment infiltration, and therapy response in colorectal cancer
Source: Cancer Immunol Immunother. 2024 Jun 4;73(8):156. doi: 10.1007/s00262-024-03731-4 (PMC11150338; doi:10.1007/s00262-024-03731-4)
Supplement: Supplementary file 1 — Supplementary file1 (DOCX 6376 KB) [file 262_2024_3731_MOESM1_ESM.docx]

**Supplementary Figures**

**
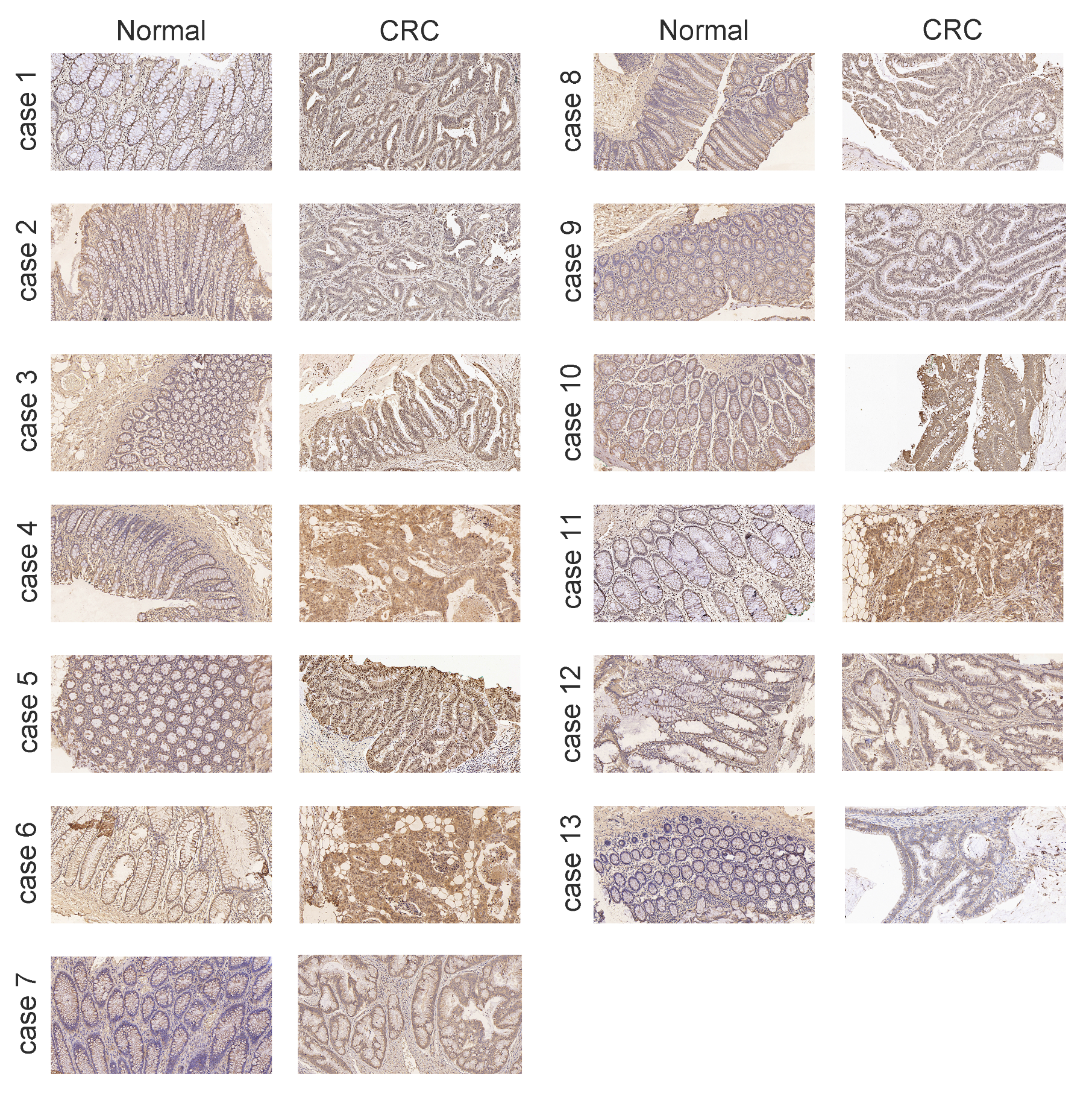
**

**Figure S1** The remaining 13 cases of USP21 expression normal colon and CRC tissues.
